# Primary Esophageal Rhabdomyosarcoma: An Exceptionally Rare Cause of Pediatric Dysphagia

**DOI:** 10.1155/crgm/3648155

**Published:** 2024-12-12

**Authors:** Maryam Ataollahi, Amirali Mashhadiagha, Fereshteh Karbasian, Reza Moshfeghinia, Javad Arabpour, Bita Geramizadeh

**Affiliations:** ^1^Department of Pediatric Gastroenterology, Shiraz University of Medical Sciences, Shiraz, Iran; ^2^Shiraz Transplant Research Center, Shiraz University of Medical Sciences, Shiraz, Iran; ^3^Department of Pediatric Gastroenterology and Hepatology, Ali-Asghar Children's Hospital, Iran University of Medical Sciences, Tehran, Iran; ^4^Student Research Committee, Shiraz University of Medical Sciences, Shiraz, Iran; ^5^Research Center for Psychiatry and Behavior Sciences, Shiraz University of Medical Sciences, Shiraz, Iran; ^6^Substance Abuse Research Center, Shiraz University of Medical Sciences, Shiraz, Iran; ^7^Young Researchers and Elite Club, Tehran Medical Science, Islamic Azad University, Tehran, Iran

**Keywords:** cancer, dysphagia, esophagus, pediatrics, rhabdomyosarcoma

## Abstract

**Background:** Esophageal embryonal rhabdomyosarcoma (ERMS), a rare pediatric cancer, mimicked achalasia in a case involving dysphagia and vomiting. Diagnosis and chemotherapy necessitate careful monitoring due to potential complications.

**Case presentation:** A 12-year-old girl with no prior medical history presented with progressive dysphagia and vomiting. Initial diagnosis suggested achalasia, but further evaluation revealed a large mediastinal mass causing esophageal compression. Biopsies confirmed primary ERMS of the esophagus with metastases. Despite chemotherapy, she developed complications, including neutropenic enterocolitis and posterior reversible encephalopathy syndrome (PRES). Unfortunately, she succumbed to neutropenic sepsis.

**Conclusion:** In this case study, we presented our experience regarding the clinical course of this disease, treatment strategy, and prognosis, in addition to the limited previous information in the literature.

## 1. Introduction

Rhabdomyosarcoma (RMS), a malignant tumor of mesenchymal origin, is the third most common extracranial malignant solid tumor in children and adolescents [1]. Although a rare disease, RMS is a reasonably common form of childhood cancer and is the most common soft tissue sarcoma in children. The overall incidence rate of RMS is approximately 4.5 patients per million individuals aged < 20 years [2, 3]. This case report describes the clinical course of a 12-year-old girl with embryonal RMS (ERMS) of the esophagus and liver.

The patient initially presented with progressive dysphagia and intractable vomiting thought to be due to achalasia. However, further investigations, including an endoscopy and barium swallow tests, revealed a mass in the esophagus that was eventually diagnosed as ERMS, the most common subtype of RMS. CT scan showed a sizable lobular mass in the posterior mediastinum and multiple hypodense lesions in both liver lobes, suggestive of metastatic disease.

The patient underwent pneumatic balloon dilatation due to achalasia but was switched to chemotherapy with vincristine and irinotecan due to complications. Despite receiving G-CSF, the patient developed complications, including neutropenic enterocolitis and posterior reversible encephalopathy syndrome (PRES), which required prompt management.

Although chemotherapy is the primary treatment for RMS, it can cause severe side effects, especially in patients with advanced disease. Therefore, carefully monitoring and managing treatment-related complications are essential to ensure the best patient outcome [4].

In conclusion, ERMS of the esophagus and liver is a rare and aggressive malignancy that can pose significant diagnostic and management challenges [5]. The case presented here highlights the importance of considering RMS in the differential diagnosis of patients presenting with dysphagia and vomiting, even in younger patients [6, 7]. It also emphasizes the need to monitor and manage treatment-related complications in chemotherapy patients carefully [5]. Early diagnosis and prompt management of RMS are critical to ensure the best possible outcome for patients, especially in cases of metastatic disease [5, 8].

## 2. Case Presentation

**Figure 1:**
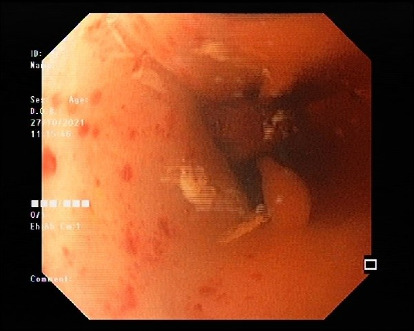
Barium swallow test. Body of esophagus dilated and has stricture of the lower sphincter bird's beak.

**Figure 2:**
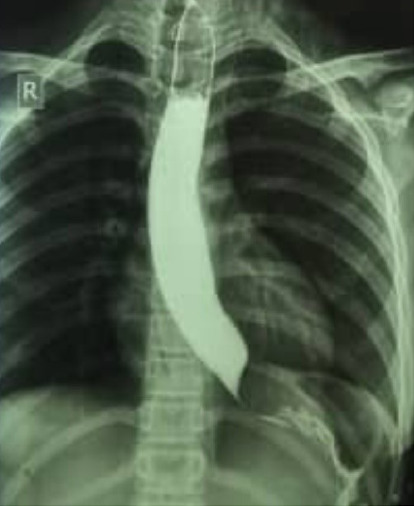
Upper endoscopy. Lower oesophageal sphincter stricture.

A 12-year-old girl with an unremarkable medical history was referred to a pediatric gastroenterology clinic due to worsening dysphagia and persistent vomiting after swallowing. Her symptoms initially began with solid food and progressed to liquids over 2 months before her first hospital admission on October 20, 2021, when she became unable to swallow anything. The first endoscopy on October 17, 2021, suggested a possible diagnosis of achalasia (Figure 1). During the endoscopic examination, a stricter lower esophageal sphincter and a mildly dilated esophagus containing residual materials were observed. A Barium swallow test on October 18, 2021, confirmed a bird's beak sign, supporting the diagnosis of achalasia (Figure 2). Based on clinical suspicion and radiological findings, pneumatic balloon dilation was performed during the initial admission. However, shortly after being discharged, she was readmitted due to loss of consciousness and diagnosed with vasovagal shock. Less than 5 days later, the procedure was repeated due to symptom recurrence, and the patient was considered for myotomy. During a diagnostic laparoscopy on November 2, 2021, a gastric mass with multiple metastases to the liver and peritoneum was discovered.

**Figure 3:**
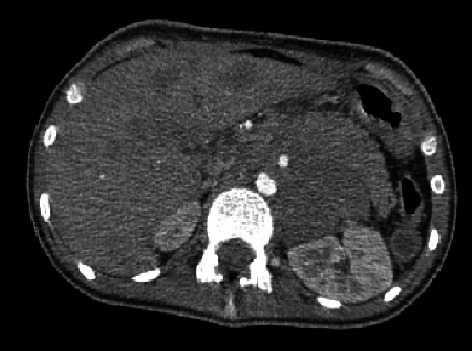
Computerized tomography (CT) scan of mass and metastasis. Large lobulated heterogeneous enhancing mass in the posterior mediastinum with multiple hypodense lesions were seen in both hepatic lobes, suggestive of the metastatic lesion.

Additionally, a liver biopsy and jejunostomy tube insertion were performed during this operation. Computerized tomography (CT) scan revealed a large lobulated heterogeneous enhancing mass in the posterior mediastinum measuring 100∗97 mm with extended to the abdominal cavity that causing pressure effect over the distal part of the esophagus, encasement of the abdominal aorta and superior branch, celiac trunk, splenic artery, and vein, portal vein, and left renal vein. Also, multiple hypodense lesions were seen in both hepatic lobes, suggestive of metastatic lesions with periportal edema. Also, several para-aortic and mesenteric lymph nodes, the most extensive measuring about 12.5 mm (Figure 3).

**Figure 4:**
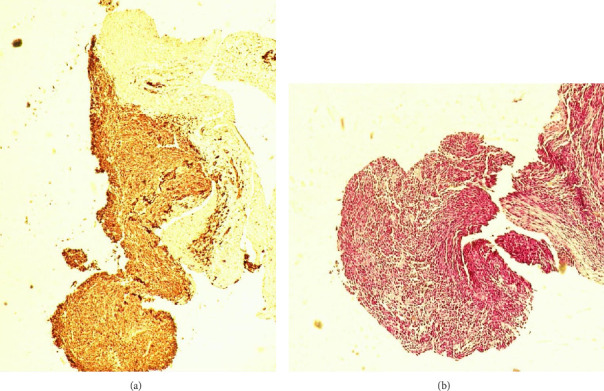
Smear of esophageal mass biopsy.

The pathology report confirmed liver metastasis through positive desmin (D33), myogenin, and a high Ki67 (MIB-1) index. It exhibited isolated and infrequent clusters of atypical cells, characterized by a high nucleus-cytoplasm (N/C) ratio, irregular nuclear borders, and numerous mesothelial cells alongside mature and immature lymphocytes. These findings collectively supported the diagnosis of ERMS. Additionally, biopsies of the esophageal mass were conducted, revealing the presence of primary (embryonal) RMS in the esophagus (Figures 4(a)(a) and 4(b)).

Due to the advanced stage of the disease, a chemotherapy regimen was selected, which involved administering a single dose of Vincristine followed by 5 days of Irinotecan. Despite receiving the prescribed doses of granulocyte-colony stimulating factor (G-CSF), the patient was readmitted 10 days after discharge with suspicion of neutropenic enterocolitis (typhlitis), as indicated by sonography. In response, empirical treatment was initiated with intravenous Piperacillin/Tazobactam and Vancomycin.

Following multiple seizures that occurred after the initial chemotherapy session on December 8, 2021, a brain CT scan was performed. The scan identified bilateral involvement of the frontal, high parietal, and occipital lobes, displaying increased signal intensity in cortical and subcortical regions. Some cortical areas exhibited restricted diffusion without enhancement. Additionally, both cerebral hemispheres showed heightened signal intensity without enhancement or restriction. Furthermore, the lateral deep gray matter, particularly the bilateral caudate nucleus, displayed increased signal intensity without enhancement or limitation. These findings raised the suspicion of PRES, and subsequently, she developed hepatic encephalopathy.

During the last sonography on December 21, 2021, it was observed that the most significant lesion among multiple hypoechoic liver lesions measured 20 mm, indicating metastasis. Additionally, several aggregated heterogeneous mass-like lesions were noted in the epigastric area near the celiac trunk, pancreas, and left liver lobe. Furthermore, there was no evidence of metastasis in the bone biopsy report from November 30, 2021. The patient presented with renal failure, electrolyte imbalance, and azotemia. Thrombosis and any pressure caused by the mass on the renal vessels were ruled out. Unfortunately, the patient ultimately passed away due to neutropenic sepsis.

## 3. Discussion

In this case report, we presented a 12-year-old girl with primary esophageal ERMS who presented with progressive dysphagia and intractable vomiting. RMS is traditionally classified into two major histological subtypes, ERMS and alveolar RMS (ARMS); 60%–70% of cases are ERMS, and 20%–30% are ARMS [9]. The patient's initial symptoms were consistent with achalasia, a condition commonly seen in children and adolescents, which suggested the need for further investigation. However, subsequent imaging and biopsy revealed the presence of a sizable esophageal mass with metastasis to the liver and peritoneum, confirming the diagnosis of ERMS [10].

The diagnosis of ERMS is challenging due to its rarity and nonspecific clinical presentation. In this case, the diagnosis was confirmed by histopathological and immunohistochemical analysis of biopsy specimens, which showed the presence of characteristic markers such as desmin, myogenin, and Ki67 [6, 11]. These markers are widely used to diagnose RMS and have high sensitivity and specificity [12]. Immunohistochemistry plays a critical role in diagnosing ERMS, and its use can help distinguish it from other types of tumors.

In cases presenting with dysphagia and esophageal masses in children, several conditions must be considered as differential diagnoses. While ERMS is rare, initial symptoms may overlap with other esophageal or mediastinal conditions, which require a systematic approach to diagnosis [13, 14].

Achalasia, a more common cause of dysphagia in pediatric patients, was initially suspected in this case due to the presentation of dysphagia and the bird's beak sign on barium swallow imaging. However, achalasia typically lacks the presence of a mediastinal mass on imaging, which was later observed in this patient. Additionally, biopsy and immunohistochemistry for markers like desmin and myogenin, which are specific to RMS, are crucial in differentiating ERMS from benign esophageal strictures or achalasia [15, 16].

Another differential diagnosis includes other types of sarcomas or lymphomas that can occur in the mediastinum or esophagus, presenting similarly with mass effect and compression symptoms. For example, lymphoma may cause a large mediastinal mass with associated systemic symptoms but would differ in immunophenotype from ERMS and lack specific myogenin expression. Thorough imaging, including CT and MRI, alongside histopathological analysis, are essential in distinguishing ERMS from these conditions, as these tumors are highly aggressive and carry a poor prognosis if misdiagnosed [17, 18].

This case emphasizes the importance of considering rare malignancies such as ERMS in the differential diagnosis of pediatric dysphagia, especially when initial findings suggest more common conditions like achalasia.

The treatment of ERMS is multimodal and is based on the extent and location of the tumor, as well as the age and general health of the patient. In this case, the patient underwent chemotherapy with Vincristine and Irinotecan, a typical treatment regimen for ERMS [7]. Despite receiving the prescribed doses of G-CSF, the patient was readmitted with an impression of neutropenic enterocolitis (typhlitis) and was treated with antibiotics. She also developed hepatic encephalopathy, renal failure, and electrolyte imbalances, which are known complications of chemotherapy. Additionally, she experienced multiple seizures after the first round of chemotherapy, which led to a brain CT scan that revealed the possibility of PRES.

The development of PRES is a rare complication of chemotherapy and is associated with hypertension, renal failure, and immunosuppressive therapy [7]. The pathophysiology of PRES is not well understood, but it is thought to be related to cerebral edema and blood-brain barrier dysfunction. In this case, the patient's PRES was likely related to her chemotherapy regimen and underlying metastatic disease. This highlights the need for close monitoring and management of chemotherapy-related toxicities in patients with ERMS.

The prognosis of ERMS depends on the extent and location of the tumor, as well as the age and general health of the patient. In this case, the patient had metastasis to the liver and peritoneum, which is associated with a poor prognosis. Despite undergoing chemotherapy, she ultimately succumbed to neutropenic sepsis. This underscores the need for more effective treatment options for patients with advanced-stage ERMS and the importance of early detection and prompt treatment [6, 19].

Moreover, this case report highlights the importance of a multidisciplinary approach to managing patients with rare malignancies such as ERMS. The involvement of a team of specialists, including pediatric oncologists, radiologists, pathologists, and surgeons, can help ensure accurate diagnosis, appropriate staging, and timely treatment. In addition, close collaboration between healthcare providers can help identify and manage chemotherapy-related toxicities and improve patient outcomes.

Finally, this case report underscores the need for further studies to understand better the epidemiology, pathophysiology, and treatment options for ERMS. Given its rarity, large-scale studies are needed to determine the optimal treatment for patients with ERMS, particularly those with advanced-stage disease. In addition, further research is required to identify biomarkers that can aid in the diagnosis and prognosis of ERMS and to develop targeted therapies to improve patient outcomes.

## 4. Conclusions

In conclusion, this case report offers valuable insights into diagnosing, treating, and managing the uncommon malignancy ERMS. The difficulties in diagnosing and treating ERMS underscore the necessity for a multidisciplinary approach and vigilant monitoring of chemotherapy-related side effects. Additional research is imperative to advance more efficient treatment modalities for individuals with advanced-stage ERMS and to enhance our comprehension of the pathophysiology behind chemotherapy-related complications.

In conclusion, this case report highlights the importance of considering rare malignancies such as ERMS in the differential diagnosis of patients with nonspecific symptoms. The diagnosis of ERMS can be challenging, but immunohistochemistry plays a critical role in confirming the diagnosis. The treatment of ERMS is multimodal and is based on the extent and location of the tumor, as well as the age and general health of the patient. The development of chemotherapy-related toxicities such as neutropenic enterocolitis and PRES underscores the need for close monitoring and management of patients with ERMS. Further research is needed to develop more effective treatment options for patients with advanced-stage ERMS and better understand the pathophysiology of chemotherapy-related complications such as PRES.

## Data Availability

All information the patient requires is given in the text; other supplementary information can be obtained via email from the corresponding author.

## Nomenclature

ERMS: Esophageal embryonal rhabdomyosarcoma
PRES: Posterior reversible encephalopathy syndrome
RMS: Rhabdomyosarcoma
CT: Computerized tomography
N/C: Nucleus-cytoplasm
G-CSF: Granulocyte-colony stimulating factor
ARMS: Alveolar RMS
